# Objective Determination of Retinal Function in Bietti Crystalline Retinopathy

**DOI:** 10.4274/tjo.02693

**Published:** 2016-06-06

**Authors:** Dorukcan Akıncıoğlu, Ümit Yolcu, Abdullah İlhan, Fatih Çakır Gündoğan

**Affiliations:** 1 Gülhane Military Medical Academy, Department of Ophthalmology, Ankara, Turkey; 2 Siirt Military Hospital, Ophthalmology Clinic, Siirt, Turkey; 3 Erzurum Military Hospital, Ophthalmology Clinic, Erzurum, Turkey

**Keywords:** Bietti crystalline dystrophy, electroretinography, multifocal electroretinography

## Abstract

A 44-year-old female patient without any known systemic or ocular disease presented with progressive visual loss and night vision disturbance. Visual acuity was 0.6 in the right eye and 0.2 in the left eye. Tiny, yellow crystalline deposits were seen on fundus examination. In addition, areas of retinal pigment epithelium and choriocapillaris atrophy were detected. Rod and cone responses were depressed in full-field flash electroretinogram. Multifocal electroretinogram testing showed severe foveal function disturbance with less severe but still depressed responses toward the periphery. Multiple hyperreflective lesions were detected in the retina in optical coherence tomography. We aimed to present the role of ocular electrophysiology by comparing the patient’s signs and symptoms with her ocular electrophysiological test results.

## INTRODUCTION

Bietti crystalline corneoretinal dystrophy (BCD) is a rare disorder characterized by yellow deposits in the retina and progressive atrophy of the retinal pigment epithelium (RPE) and the choriocapillaris. Bietti first described corneal crystals in 1937 in a report of three cases exhibiting shiny yellow retinal deposits and RPE atrophy. Lipid aggregates resulting from abnormal lipid metabolism form these crystal deposits, which may also be seen in the conjunctiva, fibroblasts and lymphocytes.^1,2^ Although some cases have crystals in the paralimbal superficial corneal stroma, corneal depositions are not typical and corneal involvement is not one of the diagnostic criteria.^3^ In BCD, symptoms including declining visual acuity and disruptions in night and peripheral vision emerge in the third and forth decades.^1^ Recent studies have demonstrated that the condition is associated with mutations in the CYP4V2 gene on chromosome 4q35.^4,5,6^ CYP4V2 encodes the cytochrome P450 enzyme group; mutations in this gene result in lipid metabolism dysfunction at the cellular level.^7^ Histopathologic studies have revealed complex lipid inclusions and crystals in the choroidal fibroblasts as well as generalized choroidal atrophy.^2^

With this case report, we aimed to discuss multifocal electroretinogram (mfERG) and electroretinogram (ERG) findings in BCD.

## CASE REPORTS

**Figure 1:**
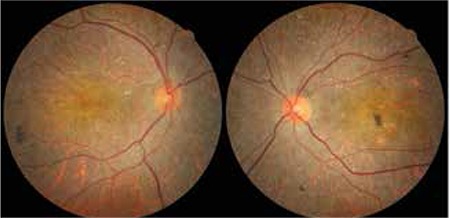
Fundus images from the patient

A 44-year-old female patient presented complaining of progressive decline in visual acuity over the previous 6 years. Her history indicated no chronic conditions or medication use. Her best corrected visual acuity was 0.6 OD and 0.2 OS; intraocular pressure was 13/11 mmHg OD/OS. Slit-lamp biomicroscopic examination was within normal limits. Small, yellow, shiny punctate intraretinal deposits were observed in the posterior pole and midperipheral retina of both eyes on fundus examination. Bilateral local RPE and choriocapillaris atrophy and pigment clusters were also observed on fundus examination (Figure 1).

**Figure 2:**
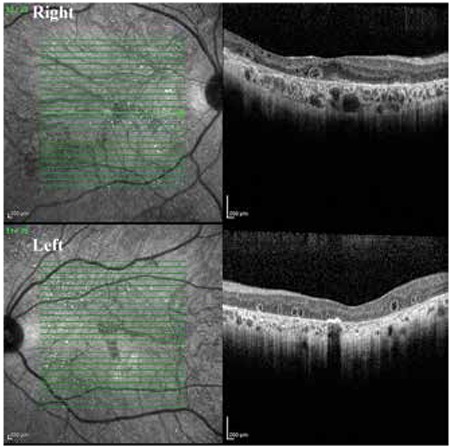
Optical coherence tomography images from each eye

Optic coherence tomography (OCT) revealed multiple hyperreflective punctate lesions in the inner and outer retinal layers with multiple cystic alterations in the outer retinal layers (Figure 2). These alterations did not correspond directly to the areas of crystalline deposits, and there were sporadic cystic alterations in the choroidal layer which did not correspond to the cystic lesions in the retina. The lesions were not present in areas of fibrosis. In OCT cross-sections the hyperreflective retinal deposits are usually found at the RPE/choriocapillaris junction and in the choroid, and sometimes in the inner retinal layers.^8,9^

**Figure 3:**
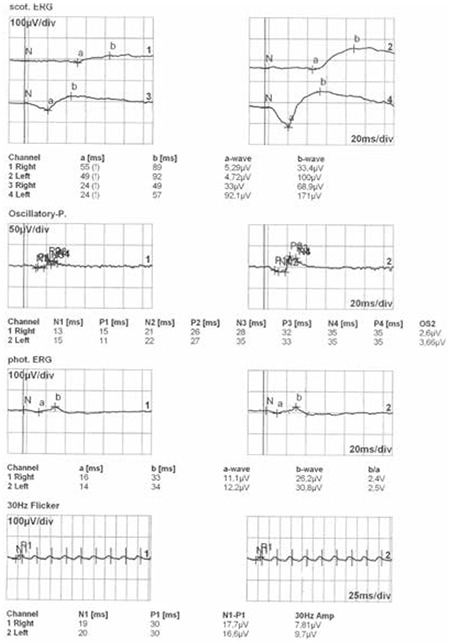
The patient’s full-field electroretinogram

According to the normal full-field electroretinography values in our clinic,10 the patient’s b-wave amplitude on dark-adaptated 0.01 ERG (rod response) was below the normal limit in the right eye (33.4 µV) but was within normal range in the left eye (100 µV) (normal range: 47-124 µV). On dark-adapted 3.0 ERG, both a- and b-wave implicit times were prolonged in both eyes, a-wave amplitudes were reduced in both eyes but the reduction was more pronounced in the right eye, and b-wave amplitude was reduced in the right eye. Light-adapted 3.0 ERG (cone response) revealed normal a- and b-wave implicit times, but both a- and b-wave amplitudes were below the normal limit. On light-adapted 30 Hz flicker ERG, P1 peak time was prolonged and the 30 Hz amplitudes were attenuated (Figure 3).

**Figure 4:**
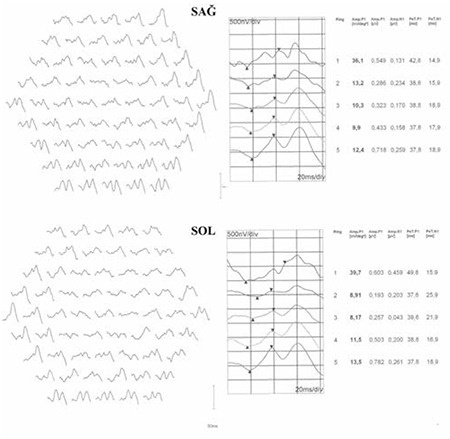
The patient’s multifocal electroretinogram in right (top) and left (bottom) eyes

Multifocal ERG revealed depressed foveal responses in both eyes; retinal function improved moving from the center toward the periphery, but the amplitudes were still markedly attenuated compared to values from normal individuals (Figure 4).^11^

## DISCUSSION

Our patient’s symptoms included bilateral progressive vision loss and more recently, difficulty distinguishing figures in the dark. Studies in the literature concerning BCD have reported pathologic ERG responses at non-detectable levels,^1,12,13^ a- and b-wave amplitude attenuation on scotopic strong flash ERG,^14^ reduced rod b-wave amplitude,^1,15^ attenuated a- and b-wave amplitudes on photopic ERG,^15,16^ decreased 30-Hz flicker amplitude,^1,16^ and abnormal S-cone ERG findings.^15^ There are also studies demonstrating retinal dysfunction using focal and mfERG, despite normal ERG findings.^16,17^

Our patient’s rod b-wave amplitudes were lower than normal in the right eye and within normal range in the left eye. Attenuated amplitude and prolonged latencies were detected in both a- and b-wave combined rod-cone responses. Photopic response showed reduced amplitudes and extended peak times in both cone and 30 Hz flicker responses in both eyes. These electrophysiological findings indicate widespread outer retinal dysfunction.

MfERG represents the electrical response to cone stimulation, usually from the central 30-40 degree field of the retina, and originating mostly from bipolar cell activity. MfERG provides local retinal function data from 61, 103 or 241 retinal sectors. Our patient showed pathologic cone responses on ERG in both eyes, and mfERG revealed markedly attenuated amplitudes in both eyes, particularly in central sectors. Because BCD is a progressive condition, observing different electrophysiological responses in the the different stages of the disease is understandable. However, in patients showing normal ERG results in the early stages of the disease, retinal function can be assessed using mfERG. We believe the utilization of mfERG is especially advisable in monitoring progression in cases with central involvement as mfERG shows the function of cone cells, which are more concentrated in the central retina. Therefore, a BCD diagnosis should not immediately be ruled out in cases exhibiting normal photopic and scotopic responses on ERG. It must be kept in mind that ERG results may be within normal limits in early BCD and that local retinal dysfunction may be detected with mfERG.

In our patient, the hyperreflective areas detected on OCT did not correspond directly to the retinal crystalline deposits, whereas Ayata et al.^9^ showed these hyperreflective areas to be consistent with the crystalline deposits. Although a clear relationship between retinal crystalline deposits and visual prognosis could not be determined, patients with widespread crystalline deposits in the sensorial retina have lower visual acuity.^18^ We believe that the cystic alterations, which are more common in the outer layers, are instances of outer retinal tubulation. Zweifel et al.^19^ first described this OCT finding as the result of morphological changes occurring after damage to cells in the photoreceptor layer in retinal degenerative diseases, and determined that this finding can be seen in BCD. Pennesi et al.^20^ hypothesized that these lesions are crystalline deposits that have been encapsulated by the retinal pigment epithelium. In terms of the clinical significance of these lesions, on B-scan OCT they can be mistaken for cystic macular edema, and their tubular structure can be more clearly visualized in C-scan sections on en face OCT. We believe the alterations observed in the choroid are an optical illusion created by these tubular alterations in the outer retinal layer.

## CONCLUSION

Evaluated together with other cases presented in the literature, our mfERG and ERG findings indicate widespread dysfunction of the outer retinal layers in BCD. As mfERG demonstrates central retinal function, this technique can be utilized as an objective assessment method when following macular function in BCD.

## Ethics

Peer-review: Externally peer-reviewed.
